# Retrospective analysis of whole blood donor demographics at a rural University in India

**DOI:** 10.6026/973206300210952

**Published:** 2025-05-31

**Authors:** Arvind Kumar Singh, Aaditya Shivhare, Yatendra Mohan, Jyoti Kala Bharati, Nouratan Singh

**Affiliations:** 1 Department of Transfusion Medicine, Uttar Pradesh University of Medical Sciences, Saifai, Etawah, Uttar Pradesh, India

**Keywords:** Blood donation, donor demographics, voluntary blood donation, rural blood donors

## Abstract

The WHO highlights a significant disparity in blood donation, with developing countries, comprising 82% of the global population, contributing only 39% of the world's blood supply. Addressing this gap requires promoting voluntary blood donation and understanding donor demographics. This study examines the demographic profile of blood donors in rural Uttar Pradesh (UP) from 2018 to 2023, analyzing data from 45,067 donors at UPUMS Blood Centre. Results show that 97.6% of donors were male, 73% were first-time donors and 87.9% were aged 18-34. Blood group distribution was "B" Positive (33.7%), followed by "O" Positive (29.4%), A Positive (26.3%) and "AB" Positive (10.6%) and 4% were Rh-ve. The findings suggest that strategies to engage women encourage repeat donations and target students and professionals could improve donor recruitment and retention.

## Background:

According to the National AIDS Control Organization (NACO), the annual blood donation rate in India is 7.4 million units, with a shortfall of 2.6 million units [1]. The WHO reports that developing countries, home to 82% of the global population, collect only 39% of the world's blood supply [2]. This highlights a significant gap between demand and supply of blood units in these regions and for a sustainable healthcare system, blood and its components play a vital role. Donated blood is crucial for treating various conditions, such as surgical blood loss, trauma, severe anemia, hematological malignancies and pregnancy-related complications [3]. Blood collection services must ensure donations come from low-risk individuals for transfusion-transmissible diseases while safeguarding donor health [4]. Therefore, careful assessment of donor eligibility is essential to ensure the safety and adequacy of the blood supply, protecting both recipients and donors while minimizing unnecessary deferrals [5]. Blood donation rates vary significantly across countries, influenced by socioeconomic factors. For example, whole blood donation rates per 1,000 populations per year were 32.1 in high-income countries, 14.9 in upper-middle-income countries, 7.8 in lower-middle-income countries and 4.6 in low-income countries [6]. Blood donors are typically categorized into voluntary, family replacement, remunerated and autologous donors. Family replacement donors donate on behalf of hospitalized family members, friends, or associates [7]. Among these, voluntary donors-those who donate altruistically and are aware of the risks-are considered the safest [8]. Understanding donor demographics and characteristics is crucial for developing effective recruitment and retention strategies, improving the efficiency and safety of blood collection efforts [9]. For example, prior studies have found that the age distribution of donors varies across regions, with younger age groups contributing the most donations in some areas, while older individuals dominate in others [10]. Similarly, gender distribution varies, with some studies reporting higher donation rates among men, while others suggest the opposite [11, 12, 13-14]. This study aims to analyse the demographic profile of blood donors at UPUMS through a five-year retrospective analysis. Therefore, it is of interest to evaluate the trends and patterns in blood donation based on the collected demographic data, providing insights into donor characteristics and informing future blood donation campaigns in the rural region of Uttar Pradesh.

## Materials and Methods:

This population-based retrospective study was conducted at the Blood Centre of Uttar Pradesh University of Medical Sciences (UPUMS), a tertiary care hospital located in the rural part of Uttar Pradesh, India. The study focused on a five-year period, analysing blood donation records from 2019 to 2023. The socio-demographic variables considered in this study included age, gender (male and female), occupation (classified as students, healthcare workers, professionals and others), educational level (primary, secondary, graduate, postgraduate), frequency of donation (first-time or regular donors) and socio-economic condition of the whole blood donors. A structured questionnaire was used to collect information on these sociodemographic characteristics in relation to blood donation. This data was sourced from the whole blood donor records maintained at the UPUMS Blood Centre in Saifai, Etawah and Uttar Pradesh. Statistical analyses were performed using SPSS software (version 22). The chi-square test was applied to determine the significance of observed differences between demographic groups.

## Inclusion criteria:

[1] All whole blood donors who visited the UPUMS Blood Bank.

[2] Donors who completed the full donation process (screening, donation and post-donation care).

## Exclusion criteria:

Donors who failed to meet the basic eligibility criteria.

## Results:

A majority of blood donors were replacement donors (88%) and only 12% donors were of voluntary categories respectively shown in Table 1 and Figure 1 - (see PDF). These findings indicate that this is need of awareness and motivational strategies in this area to increase in voluntary blood donation at UPUMS. A majority of blood donors were in the 18-30 age group, comprising 58% of all donations. The 31-40 age groups represented 22%, while donors aged 41-50 and those above 50 years contributed 12% and 8%, respectively (Table 2). These findings indicate that young adults are the most active age group in whole blood donation at UPUMS. In terms of donation frequency, 73% of the donors were first-time donors, while 27% were regular donors, having donated blood more than once during the study period. This suggests that there is a significant potential to retain donors and targeted strategies could be employed to encourage repeat donations shown in Table 3. The most common blood group in the present study was "B" (33.7%), followed by "O" (29.4%), "A" (26.3%) and "AB" (10.6%). About 96% reported Rhesus antigen positive and 4% were Rh-ve, shown in Figure 2 - (see PDF). A significant gender disparity was observed, with males accounting for 97.6% of donations and females contributing only 2.4% shown in Figure 3 - (see PDF). Among the study population, 45% of blood donors were students, making them the largest group. Healthcare workers and professionals accounted for 25%, while individuals from other occupational categories (*e.g.*, farmers, businesspeople) represented 30% shown in Table 4. The significant participation of students may be attributed to the university's active role in promoting blood donation as part of campus life. The educational level of blood donors revealed that the majority had completed secondary education (55%). 30% of donors had a graduate degree and 10% were postgraduates. Only, 5% of donors had primary education or less shown in Table 5. These findings suggest that higher educational attainment is associated with an increased likelihood of donating blood.

## Discussion:

The results of this study highlight several trends that can inform effective donation strategies in the region. In our study, the majority of donors were men (97.6%) compared to women (2.4%). These findings differ from studies by Al Shaer *et al.* who reported 95.89% male and 4.1% female donors [15], Birjandi *et al.* who reported 95.6% male and 4.4% female donors [16] and Unnikrishnan *et al.* who reported 95.13% male and 4.8% female donors [17]. The low percentage of female donors can be attributed to factors such as ignorance, fear, lack of awareness, high prevalence of anemia, low socioeconomic status and limited education and motivation among women in rural populations. In India, gender disparity in blood donation is well-documented, with cultural factors, physical challenges (*e.g.*, lower hemoglobin levels) and socio-economic status often discouraging female participation [18]. Targeted campaigns to raise awareness among women and improve access to donation sites could help address this issue. Our study found that the majority of blood donors were in the 18-30 age group (58%), followed by the 31-40 age group (22%), while donors aged 41-50 and above 50 years contributed 12% and 8%, respectively. These findings differ from studies in the southern part of India, where the 30-39 age groups was most common among whole blood donors [19]. Malhotra *et al.* reported that 93.8% of male and 6.2% of female donors were less than 35 years of age [20]. The dominance of younger age groups in blood donation is consistent with trends where students and young professionals are more likely to participate in voluntary donation campaigns [21].

Educational outreach programs targeting young adults in universities could further enhance donation rates. Replacement donations (88%) were significantly higher than voluntary donations (12%) in our study, likely due to fewer outdoor voluntary donation camps during the COVID-19 pandemic and limited awareness of blood donation in rural populations. A study by Kapoor *et al.* (1995-96) at G.B. Pant Hospital, New Delhi, reported that 58% of donors were replacement donors, followed by 39.3% voluntary donors [22]. Similarly, Arya *et al.* (1993-2003) from S.P. Medical College, Bikaner, found that 92.5% of blood units were collected from replacement donors, while 7.5% were from voluntary donors [23]. The most common blood group in our study was B (33.7%), followed by O (29.4%), A (26.3%) and AB (10.6%). Approximately 96% of donors were Rh-positive and 4% were Rh-negative. These findings align with studies by Garg *et al.* in Maharashtra [24], Chandra and Gupta in North India [25], Singh *et al.* [26], Kaur *et al.* [27] and Haldar *et al.* [28]. Our study revealed that most blood donors were students, followed by healthcare professionals, farmers and businessmen. The significant participation of students and healthcare professionals suggests that motivational activities conducted by various programs played a key role in donor recruitment, as most activities were concentrated in institutional organizations. Regarding educational qualifications, the majority of donors had completed secondary education (55%), followed by graduates (30%). These findings are consistent with those of Kumaran *et al.* who reported that most whole blood donors had education below the 10th standard [18]. The lower educational qualifications of donors in this rural population may reflect the socio-economic and educational landscape of rural North India. Finally, the high percentage of first-time donors (73%) compared to repeat donors (27%) underscore the need for strategies to retain regular donors. Improving donor engagement through follow-up systems could help address this gap.

## Conclusion:

A predominance of young, male and educated blood donors at UPUMS University is shown. Promoting gender balance and diversity through targeted awareness programs is essential for a sustainable blood supply. Continued research and understanding donor motivations are key to improving voluntary donations and retention, reducing societal fears and ensuring a reliable donor pool.

## Figures and Tables

**Table 1:** Type of blood donors

**Type of Donation**	**Percentage of Donors (%)**
Voluntary	88
Replacement	12
Total	100

**Table 2:** Age group distribution of blood donors

**Age Group (Years)**	**Number of Donors**	**Percentage**
18-30	26,138	58
31-40	9,915	22
41-50	5,409	12
>50	3,605	8
Total	45,067	100

**Table 3:** Frequency of blood donation

**Type of Donor**	**Number of Donors**	**Percentage (%)**
First-time Donor	32,888	73
Repeat Donor	12,179	27
Total	45,067	100

**Table 4:** Occupational profile of blood donors

**Occupation**	**Number of Donors**	**Percentage (%)**
Healthcare Workers and Other Professionals	11,266	25
Students	20,280	45
Farmers	9,914	22
Service Sector	3,605	8
Total	45,067	100

**Table 5:** Educational status of blood donors

**Education Level**	**Number of Donors**	**Percentage (%)**
Primary	2,253	5
Secondary	24,745	55
Graduate	13,551	30
Postgraduate	4,518	10.2
Total	45,067	100
